# Identification of BACH1-IT2-miR-4786-Siglec-15 immune suppressive axis in bladder cancer

**DOI:** 10.1186/s12885-024-12061-8

**Published:** 2024-03-11

**Authors:** Xingzhi Li, Ziji Liang, Jiexin Pan, Meng Zhang, Jinli Liu, Rong Hu, Caiyan Liao

**Affiliations:** 1 Department of Urology, The Second Affiliated Hospital of The Chinese University of HongKong/Longgang District People’s Hospital of Shenzhen, Shenzhen, 518172 Guangdong China; 2 Warshel Institute for Computational Biology, The Chinese University of Hong Kong, Longgang District, Shenzhen, 518172 Guangdong China

**Keywords:** Bladder cancer, BACH1-IT2, Siglec-15, miR-4786, Immune suppression

## Abstract

The sialic acid binding Ig like lectin 15 (Siglec-15) was previously identified as tumor immune suppressor gene in some human cancers with elusive molecular mechanism to be elucidated. The continuous focus on both clinical and basic biology of bladder cancer leads us to characterize aberrant abundance of BACH1-IT2 associating with stabilization of Siglec-15, which eventually contributes to local immune suppressive microenvironment and therefore tumor advance. This effect was evidently mediated by miR-4786-5p. BACH1-IT2 functions in this scenario as microRNA sponge, and competitively conceals miR-4786 and up-regulates cancer cell surface Siglec-15. The BACH1-IT2-miR-4786-Siglec-15 axis significantly influences activation of immune cell co-culture. In summary, our data highlights the critical involvements of BACH1-IT2 and miR-4786 in immune evasion in bladder cancer, which hints the potential for both therapeutic and prognostic exploitation.

**Supplementary Information:**

The online version contains supplementary material available at 10.1186/s12885-024-12061-8.

## Introduction

Immunotherapy represents the most promising clinical managements of kinds of human solid tumor with advance of understanding into the immunosuppressive microenvironment [1, 2]. Multiple fundamental mechanisms were acknowledged, and the complexity was increasingly elucidated involving deficiencies in immune cell infiltration, regulatory T cell expansion, tumor-associated macrophage maturation and myeloid-derived suppressive cell functions [3, 4]. The dysregulation of both immune-stimulation and immune-inhibition convergent in occurrence of immune evasion of tumor cells, which has been increasingly identified and targeted for therapeutic intervention. For instance, the well-recognized programmed death-ligand 1 (PD-L1) was selectively up-regulated by tumor associated T-lymphocytes-derived interferon-gamma, and specifically binds to programmed death-1 (PD-1) factor on T cell therefore blockades immune response [5, 6]. Multiple therapeutic antibodies and analogues were approved for many tumor types, and more were under clinical trials worldwide [7, 8]. However, the practical outcomes were only satisfied in part of cancer patients, which implied other immunosuppressive actions involved and motivated further academic pursues.

Siglec-15 is a member of the sialic acid binding Ig-like lectin family, which specifically associates with sialic acid sugar moieties on various proteins involving in immune modulations [9]. These effects are dependent on presence of immune-receptor tyrosine-based inhibition or activation motif domains. Siglec-15 was first identified by Wang et al. in 2019 as a universal immune suppressor presenting in many human cancer cells and intra-tumoral myeloid cells [10]. The mutual exclusion of PD-L1 and Siglec-15 across multiple cancers highlighted the great potential of Siglec-15 as intervention target in PD-L1-negative patient. Encouraging results from phase I clinical trial with Siglec-15 antibody, NC318, showed complete responses in non-small cell lung cancer patients resistant to PD-1 blockade, which was further under evaluation alone or in combination with PD-1 antibody in phase II [11].

Our previous study characterized aberrant overexpression of Siglec-15 in clear cell renal cell carcinoma (ccRCC) was tightly associated with non-coding LINC00973, which functioned as competing endogenous RNA (ceRNA) against miR-4786 and eventually positively modulated Siglec-15 abundance [12]. Here we reported distinct lncRNA and miR molecules involving in Siglec-15 regulation in bladder cancer and provided mechanistic insight into tumor immune suppression in this disease.

## Materials and methods

### Patients and clinical samples

A total of 100 bladder cancer patients participated in this investigation in Longgang District People’s Hospital of Shenzhen. All diagnosed patients received informed consent and none of any prior therapy at the time of enrollment. The clinicopathological features are summarized in Table 1. The ethical approval was acquired from the Hospital Committee and investigation was conducted in strict accordance with the Helsinki Declaration.

**Table 1:** Correlation between clinicopathological features and BACH1-IT2 levels

Variable	Number of cases	BACH1-IT2		*p*-value
		High	Low	
**Age (y)**				
> 70	26	15	11	0.289
≤ 70	74	58	16	
**Gender**				
Female	19	15	4	0.332
Male	81	49	32	
**Tumor size (cm)**				
< 4	37	27	10	0.021^*^
≥	63	61	2	
**Tumor invasion depth (T)**				
Tis, Ta, T1	69	42	27	0.014^*^
T2, T3 or above	31	26	5	
**TNM stage**				
0/I	58	47	11	0.011^*^
II/III/IV	42	38	4	

^*^Denotes significance

### Cell culture

Human bladder cancer cell lines HT1197, 5637, T24 (Basal/Squamous), SW780, RT4 (Luminal), UMUC-3 (Neuroendocrine-like) and hTERT-immortalized human urothelial cell line TRT-HU1 (normal control) were included in this study. All cell lines were obtained from ATCC and first authenticated by STR profiling method before use. Unless specified, all culture was maintained in RPMI-1640 medium supplemented with 10% FBS and 1% penicillin/streptomycin (Gibco).

All constructs used in this study including shRNA, Siglec-15 and BACH1-IT2 were purchased from Tsingke Bio. All microRNAs including miR-4786 mimic, inhibitor, scramble control were designed and produced by RiboBio. Lipofectamine 3000 was adopted for cell transfection in strict accordance with manufacturer’s instruction.

### Real-time PCR

The total RNA was isolated from indicated cells with TRizol reagent (Invitrogen) and cDNA species were generated with High-Capacity cDNA Reverse Transcription Kit (ThermoFisher). The quantitative analysis of BACH1-IT2 and Siglec-15 was performed with PowerUp SYBR Green MasterMix (ABI), and All-in-One miRNA qRT-PCR Detection Kit (GeneCopia) was employed for miRs quantitation with U6 serving as reference gene. The PCR thermal-cycling conditions were used as below: 95 °C for 10 min and 40 cycles at 95 °C for 3 s, at 60 °C for 30 s, and at 60 °C for 1 min. The relative expression was calculated by 2^−ΔΔCt^ method. The primers were as follows:

- GAPDH-forward: 5′-GGAGCGAGATCCCTCCAAAAT-3′;
- GAPDH-reverse: 5′-GGCTGTTGTCATACTTCTCATGG-3′;
- BACH1-IT2-forward: 5′- GACCTACCACTGCTCTGT-3′;
- BACH1-IT2-reverse: 5′- TGTCTGTCACTGCTACCA-3′;
- Siglec-15-forward: 5′- TGGAAGCGGAACAGGTAGAC-3′;
- Siglec-15-reverse: 5′-GGCTGTTGTCATACTTCTCATGG-3′;

### Immunofluorescence

HT1197 cells (control, BACH1-IT2 overexpression, miR-4786 mimic, miR-4786 inhibitor applied either alone or in combination as indicated) were seeded on cover glasses and cultured continuously until confluency approaching 30%. And gent rinse with PBS, cells were fixed by 4% PFA at room temperature for 10 min and followed by permeabilization with 0.3% Triton X-100 for 15 min. Fluorescence-labeled Siglec-15 antibody (1: 50, NB096711, Fisher) was applied overnight at 4 °C after brief blocking with 5% BSA. The cell nucleus was counterstained with DAPI solution, and all images were captured using Leica confocal microscope.

### Immunohistochemistry

Human bladder tumor tissues were prepared into 5-μm section and subjected to immunohistochemical stain with Biotin-Streptavidin HRP.

Detection System (ZSGB-BIO) following the provider’s protocol. Siglec-15 antibody was obtained from Abcam (ab198684) and 1:50 dilution was applied. Diaminobenzidine method was adopted for signal development. The nuclei were counterstained with hematoxylin for reference purpose. Representative images were acquired using a DMi8 Leica DMi8 Inverted Microscope.

### Western blotting

The indicated cells were lysed in ice-cold RIPA buffer with proteinase inhibitor cocktail (Roche) supplemented. Protein species were resolved by 10% SDS-PAGE gel and transferred onto PVDF membrane (Millipore). After blocking with 5% skim milk, both Siglec-15 and GAPDH were probed with specific primary antibodies (rabbit anti–Siglec-15, 1:1000, PA5-50759, ThermoFisher; mouse anti–GAPDH, 1:2500, sc-32233, Santa Cruz Biotechnology) at 4 °C overnight. After detection with HRP-labeled secondary antibodies (goat anti-rabbit, 1: 5000, 7074 and horse anti-mouse, 1:5000, 7076 from Cell Signaling Technology), the blots were visualized with enhanced chemiluminescence method (ECL, Millipore).

### RNA pulldown

The BACH1-IT2 (either wild-type or mutant) transcripts were prepared by in vitro transcription with T7 RNA polymerase and followed by biotin labeling (MEGAscript T7 transcription Kit, ThermoFisher) according to the manual. The RNeasy Mini Kit from Qiagen was used to recover and purify the produced BACH1-IT2. The biotin-labeled miR4786 and scramble control were ordered from RiboBio. The indicated nuclear extracts were incubated with probes at 4 °C for 1 h, and affinity magnetic beads was applied to pull-down interacting RNA species. The relative enrichment was determined by real-time PCR as described before.

### Luciferase assays

The Siglec-15 3’UTR, BACH1-IT2 transcript-fused luciferase plasmids were ordered from GeneCopoeia. The mutant constructs were generated by mutagenesis PCR method. The indicted plasmids in combination were co-transfected into 293 T cells and culture medium was collected 24 h later. The luciferase activity was determined with Secrete-Pair Luminescence Assay Kit (GeneCopoeia) following the provider’s guide.

### IL-2/TNF-α production assay

The immune/cancer cell co–culture system was employed to determine the immune response in regard to Siglec-15 expression as previously described. The Jurkat cells were first transduced with MART-I-specific 1D3 T cell receptor (TCR), and bladder cancer cells were loaded with MART-I peptides (10 ng/mL, 37 °C for 1 h). Co–culture was conducted at a ratio of 2:1 (Jurkat: cancer cells) at 37 °C. 20 μg/mL Siglec-15 antibody (ThermoFisher) was used for blocking assay. The secreted IL-2 and TNF-α were determined using Human ELISA Kit (Invitrogen) at 48 and 72 h, respectively. The detailed procedures were referred to manufacturer’s recommendations.

### Statistical analysis

We employed unpaired, two-tailed Student’s *t* test for statistical comparison using GraphPad Prism 8.0, and *p* < 0.05 was defined as significant difference.

## Results

### High-expression of BACH1-IT2 in bladder cancer

**Fig. 1:**
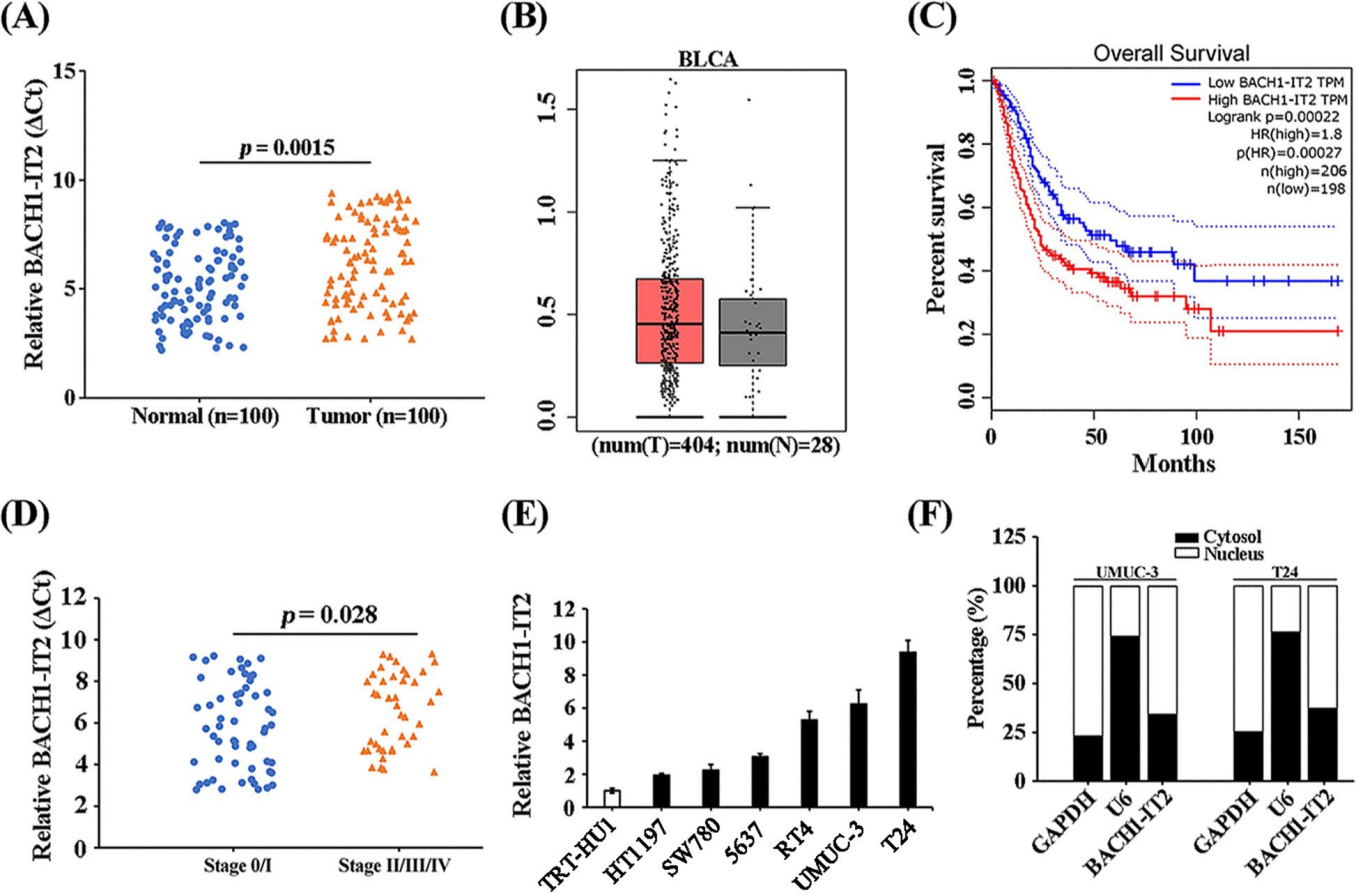
High expression of BACH1-IT2 in bladder cancer. **A** Real-time PCR quantitation of relative expression of BACH1-IT2 transcript in bladder tumors and paired adjacent normal tissues (*n* = 100, from the Longgang District People’s Hospital of Shenzhen). **B** Expression analysis of BACH1-IT2 in bladder cancers (T = 404) and normal controls (*N* = 28) in public database (Gepia). **C** Kaplan–Meier survival curve of bladder cancer patients subgrouped into high (*n* = 206) and low (*n* = 198) BACH1-IT2. **D** Correlation analysis of relative BACH1-IT2 levels with tumor stage of bladder cancer panel (*n* = 100). **E** Quantitative PCR analysis of relative expression of BACH1-IT2 in panel of bladder cancer cell lines including HT1197, SW780, 5637, RT4, UMUC3, T24 and immortalized urothelial cell line TRT-HU1. **F** Subcellular localization of BACH1-IT2 was determined by fractionization analysis with GAPDH as a cytosol marker and U6 as a nucleus marker, respectively

We first analyzed relative abundance of BACH1-IT2 in clinical bladder tumor tissues in comparison with benign normal control. As shown in Fig. 1A, significant up-regulation of BACH1-IT2 was noticed in tumor samples, which suggested the potential pro-tumoral property of BACH1-IT2 in human bladder cancer. For validation purpose, we also data-mined public database (gepia.cancer-pku.cn) and the result was in agreement with up-regulation of BACH1-IT2 in this disease (Fig. 1B). More importantly, high BACH1-IT2 obviously associated with both overall survival (Fig. 1C) and disease advance (Fig. 1D). The poor prognosis and disease develop in high-BACH1-IT2 population consolidated the pro-tumoral features of this non-coding RNA molecule. To further elucidate the biology of BACH1-IT2 in bladder cancer, we collected 6 cancer cell lines and one hTERT immortalized normal human urothelial cell line, TRT-HU1, as control. Consistent with observation on clinical samples, BACH1-IT2 was universally up-regulated in all 6 bladder cancer cell lines and the highest level of BACH1-IT2 were noticed in UMUC-3 and T24 (Fig. 1E). The subcellular quantitation suggested that majority of BACH1-IT2 transcripts was localized in cytosol other than nucleus in both UMUC-3 and T24 cells (Fig. 1F), which implicated its main biological function in this compartment.

### BACH1-IT2 upregulated Siglec-15 in bladder cancer

**Fig. 2:**
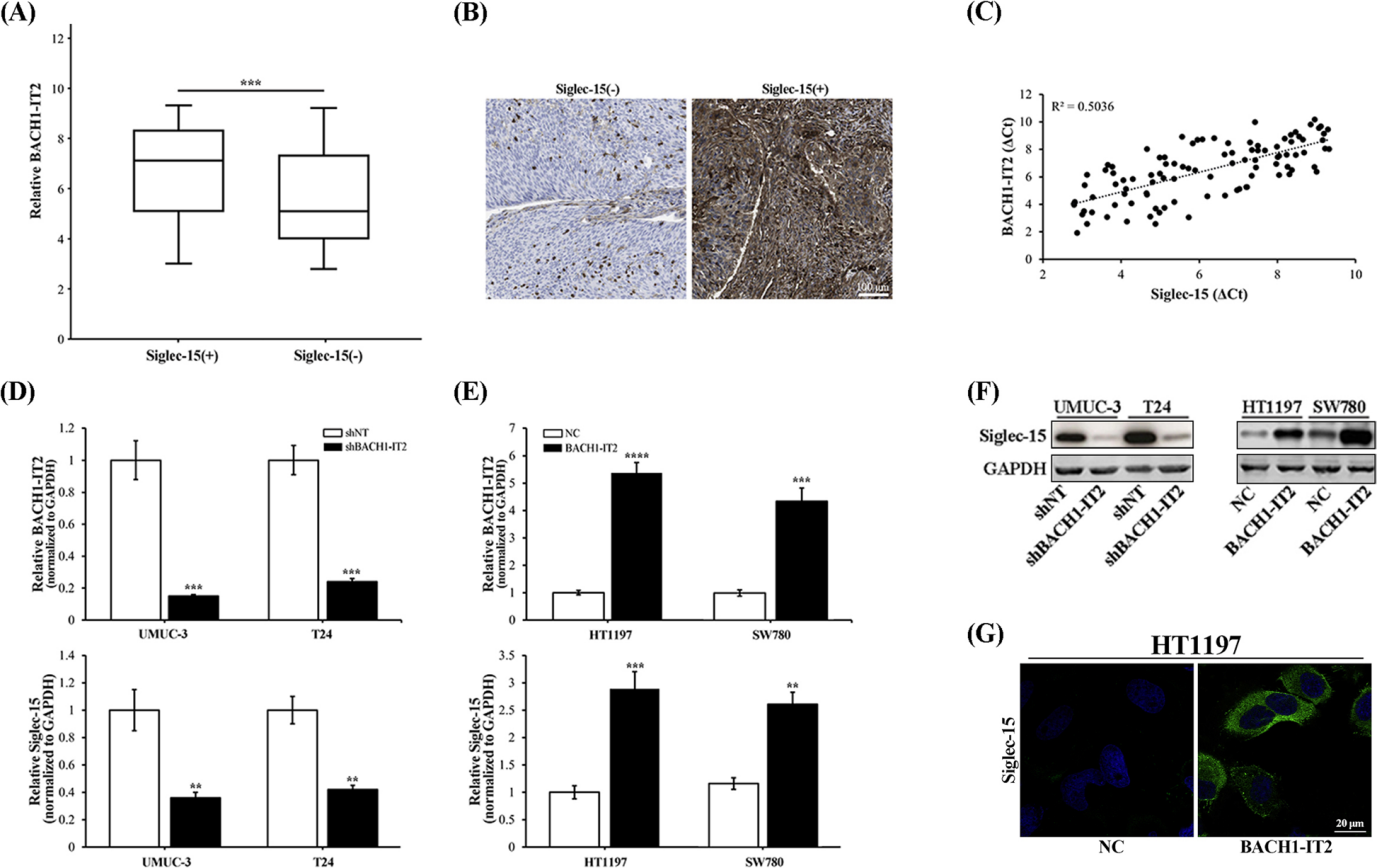
BACH1-IT2 upregulated Siglec-15 in bladder cancer cells. **A** Comparison of BACH1-IT2 abundance in Siglec-15 negative and positive bladder tumors. **B** Representative images of Siglec-15 immunohistochemistry (IHC) staining results. **C** Correlation between endogenous BACH1-IT2 and Siglec-15expression in bladder tumors. **D** Establishment of BACH1-IT2-knockdown cell lines in UMUC-3 and T24 cells was validated by real-time PCR (upper pane) and according Siglec-15 was quantified (lower pane). **E** Establishment of BACH1-IT2-overexpressing cell lines in HT1197 and SW780 cells was validated by real-time PCR and according Siglec-15 was quantified (lower pane). **F** Western blot analysis of Siglec-15 protein in BACH1-IT2 knockdown or overexpressing cells, and the croppted blots were presented correspondingly. **G** Immunofluorescence staining of cell surface Siglec-15 of HT1197 in response to either control (NC) or BACH1-IT2 overexpression

To better understand biological role of high BACH1-IT2 in bladder cancer, we sought to identify its downstream target which subjects to its predominant modulation. In this regard, we noticed significant and positive correlation between BACH1-IT2 and Siglec-15 in panel of bladder tumor, which was previously reported involving in immune-suppression of clear-cell renal cell carcinoma. The BACH1-IT2 transcripts were significantly enriched in Siglec-15-positive group (Fig. 2A). The representative IHC stain of Siglec-15 in bladder tumors was shown in Fig. 2B. Consistent with its well-recognized immune suppressor role, we also noticed that relatively low CD4 and high CD33/204 abundance associating with Siglec-15 positivity (Fig. S1). Similarly, quantitative analysis of transcript levels of Siglec-15 and BACH1-IT2 demonstrated evident correlation (Fig. 2C). To clarify whether BACH1-IT2 directly involved in Siglec-15 modulation in bladder cancer cells, we next employed shRNA to specifically knockdown BACH1-IT2 in both UMUC-3 and T24 cells. Siglec-15 transcripts were remarkably down-regulated upon BACH1-IT2 silence (Fig. 2D). In contrast, ectopic introduction of BACH1-IT2 greatly induced up-regulation of Siglec-15 (Fig. 2E). These observations were further consolidated at protein levels (Figs. 2F, S2). The direct involvement of BACH1-IT2 in Siglec-15 modulation was further intuitively demonstrated by cell immunofluorescence assay, whereas cell surface Siglec-15 was greatly intensified by ectopic overexpression of BACH1-IT2 in HT1197 cell (Fig. 2G).

### BACH1-IT2-upregulated Siglec-15 was dependent on miR-4786

**Fig. 3:**
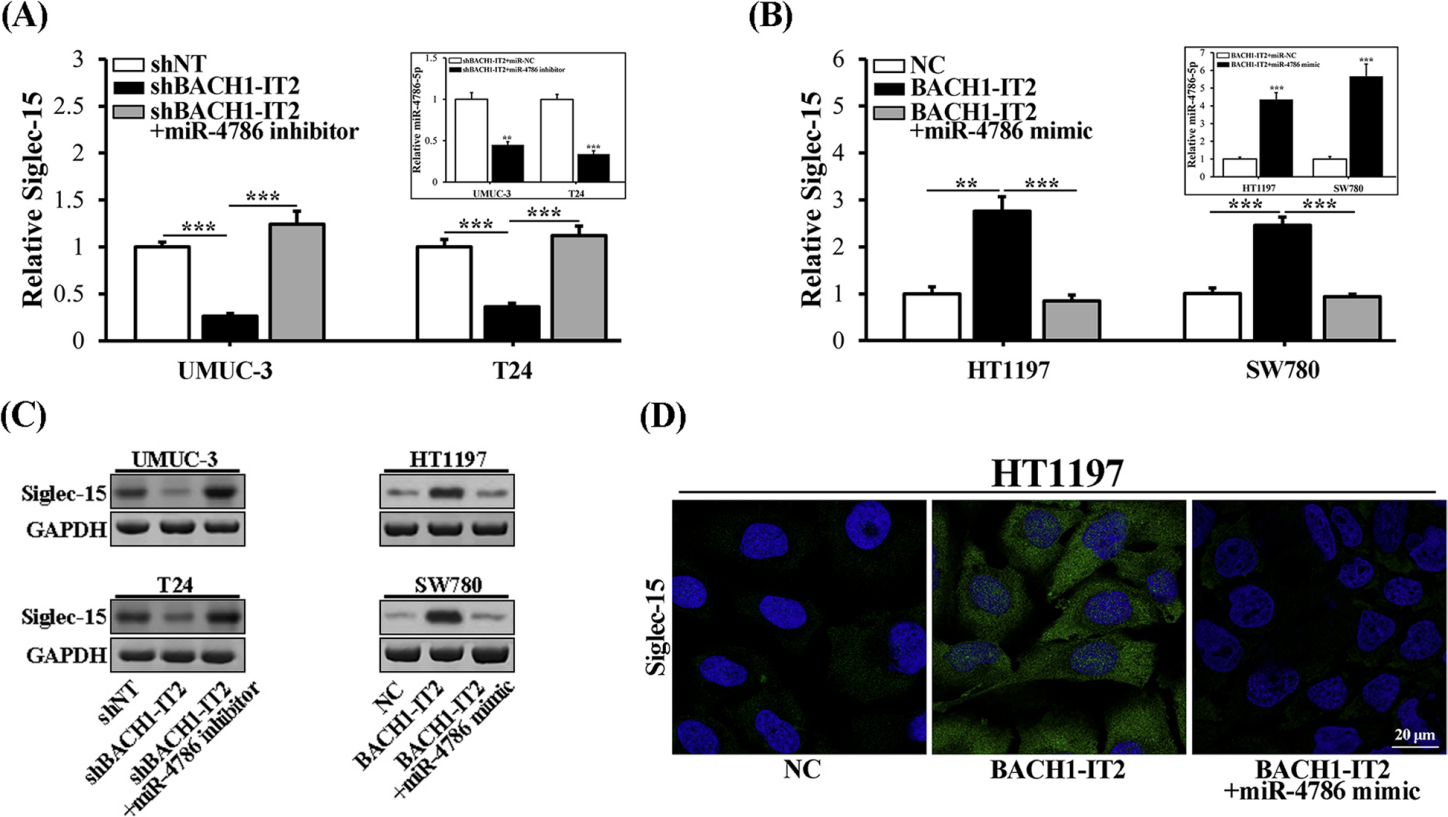
BACH1-IT2-upregulated Siglec-15 was dependent on miR-4786. **A** Quantitative analysis of Siglec-15 transcript in BACH1-IT2 intact or depleted UMUC-3 and T24 cells treated with control or miR-4786 inhibitor. Endogenous miR-4786 was quantified accordingly (upper left pane). **B** Quantitative analysis of Siglec-15 transcript in BACH1-IT2 naïve or overexpressing HT1197 and SW780 cells treated with control or miR-4786 mimics. Endogenous miR-4786 was quantified accordingly (upper left pane). **C** Western blot results of Siglec-15 protein in the cells as described in A and B, and the croppted blots were presented correspondingly. **D** Immunofluorescence staining of cell surface Siglec-15 in HT1197 cells expressing either naïve or ectopic BACH1-IT2, and transfected with either control or miR-4786 mimics

The competitive endogenous RNA was increasingly illuminated as major mode of act of variety of lncRNA [13]. Our previous study identified LINC00973-miR-7109-Siglec-15 axis played critical role in modulation of immune-suppression of ccRCC, which prompted us elucidate molecular mechanism underlying the regulation of Siglec-15 by BACH1-IT2 along this direction. Through close inspection of primary sequences of both BACH1-IT2 and 3’UTR region of Siglec-15, we speculated that miR-4786-5p as novel candidate may involve in Siglec-15 up-regulation by BACH1-IT2. To experimentally clarify this possibility, we introduced miR-4786 inhibitor in the context of BACH1-IT2 silencing in both UMUC-3 and T24 cells. Consistent with previous observation, shRNA-mediated knockdown of BACH1-IT2 greatly inhibited Siglec-15 expression, which was completely restored by co-administration of miR-4786 inhibitor (Fig. 3A). On the other hand, the tremendous induction of Siglec-15 by ectopic BACH1-IT2 was remarkably compromised by co-transfection of miR-4786 mimic (Fig. 3B). These results unambiguously suggested that miR-4786 directly and predominantly participated in Siglec-15 regulation downstream BACH1-IT2 in bladder cancer cells. This regulatory effect was also consolidated by western blotting in all subject cell lines (Fig. 3C). The cell surface Siglec-15 which was intensified by BACH1-IT2 was compromised by miR4786 mimic as well (Fig. 3D). Taken together, our data confirmed the importance of miR-4786 in regulation of Siglec-15 in response to BACH1-IT2 in bladder cancer.

### Siglec-15 was a direct target of miR-4786 in bladder cancer

**Fig. 4:**
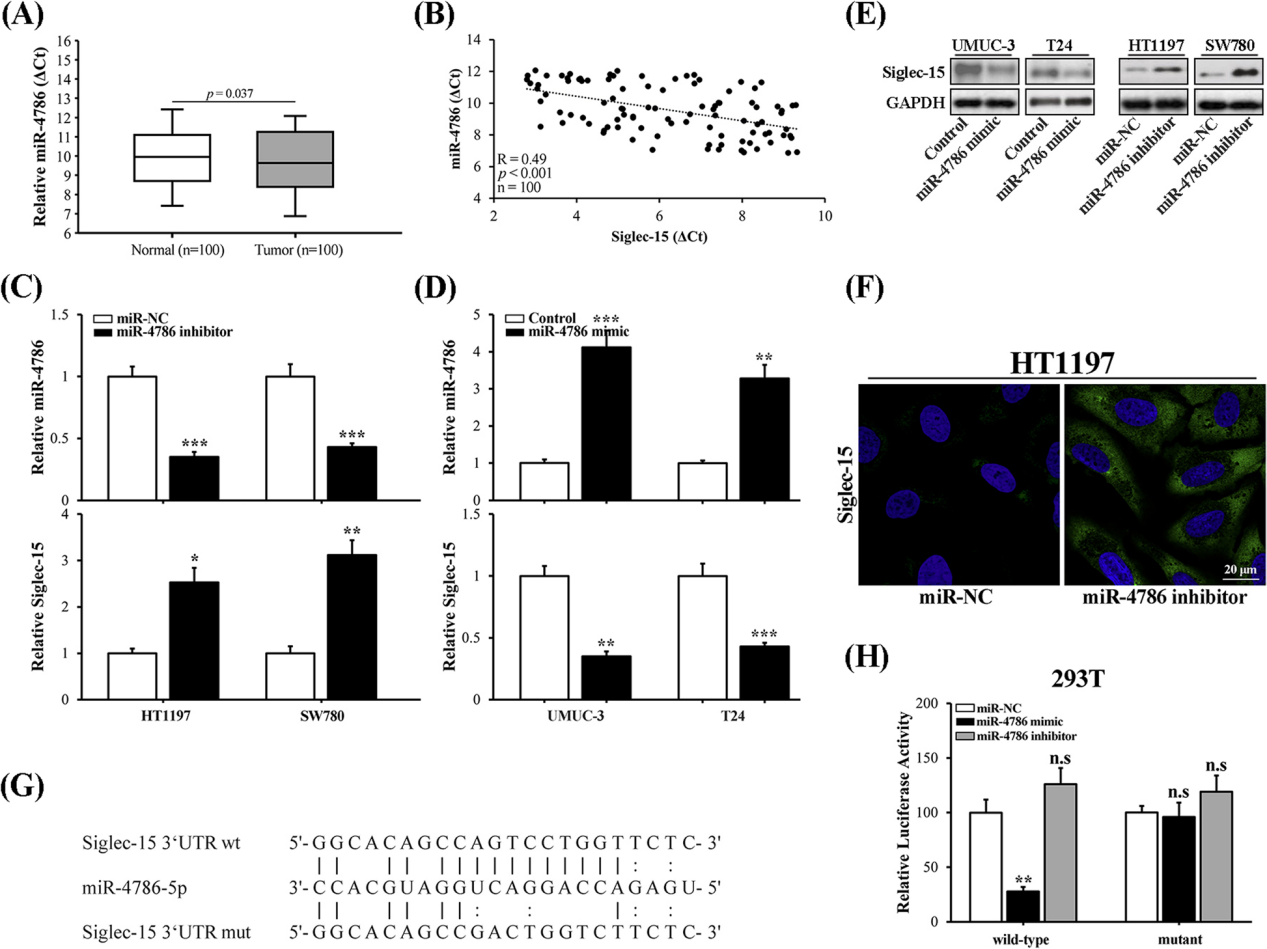
Siglec-15 was a direct target of miR-4786 in bladder cancer. **A** Real-time PCR analysis of miR-4786 expression in bladder tumor samples with paired adjacent normal tissues. **B** Correlation analysis between miR-4786 and Siglec-15 in bladder tumors (*n* = 100). **C** Change of miR-4786 expression in both HT1197 and SW780 cells in response to miR-4786 inhibitor (upper) and Siglec-15 was determined accordingly (lower). **D** Change of miR-4786 expression in both UMUC-3 and T24 cells in response to miR-4786 mimic (upper) and Siglec-15 was determined accordingly (lower). **E** Western blot analysis of Siglec-15 proteins in UMUC3 and T24 cells transfected with either control or miR-4786 mimic (left), as well as HT1197 and SW780 cells transfected with either negative control (miR-NC) or miR-4786-specific inhibitor (right), and the croppted blots were presented correspondingly. **F** Immunofluorescence staining of cell surface Siglec-15 in HT1197 cells transfected with either control or miR-4786 inhibitor. **G** Alignment between miR-4786 seed region and both wild-type and putative binding site-mutated Siglec-15 3ʹUTR. **H** Luciferase reporter analysis of the potential regulatory effects of miR-4786 on Siglec-15 in 293 T cells, which were transfected with control, miR-4786 mimics, and miR-4786 inhibitors, respectively

Next, we sought to clarify whether miR-4786 directly targeted to and modulated Siglec-15 in human bladder cancer. To this purpose, we first compared relative abundance of miR-4786 in bladder tumor and normal counterparts. The obvious suppressed expression of miR-476 was observed in tumor tissues compared to normal counterparts (Fig. 4A). The quantitative analysis of miR-4786 and Siglec-15 transcript showed significant and negative correlation in bladder tumor panel (Fig. 4B). Administration with miR-4786 inhibitor in both HT1197 and SW780 cells remarkably stimulated up-regulation of Siglec-15 (Fig. 4C). Consistently, transfection of miR-4786 mimic into both UMUC-3 and T24 cells tremendously suppressed Siglec-15 expression (Fig. 4D). The inhibitory effects of miR-4786 on Siglec-15 protein was further consolidate by either immunoblotting (Fig. 4E) and immunofluorescence (Fig. 4F). We further presented alignment between the primary sequence of miR-4786 and Siglec-15 3’UTR, the perfect match between two species was highlighted by connection lines (Fig. 4G) which may be the seed and target region. Therefore, we fused putative sequence of Siglec-15 3’UTR region with luciferase reporter gene, and potential modulation by miR-4786 in this scenario was interrogated by luciferase reporter assay. In line with previous observations, co-transfection with miR-4786 mimic into 293 T cell resulted into great inhibition of Siglec-15 3’UTR-regulated luciferase activity (Fig. 4H), while miR-4786 inhibitor imposed negligible effect. However, mutation introduced to impair recognition of Siglec-15 by miR-4786 completely abolished the inhibition. Our results presented evidence in support of direct and negative regulation of Siglec-15 by miR-4786 in bladder cancer.

### BACH1-IT2 functioned as ceRNA against miR-4786 in bladder cancer

**Fig. 5:**
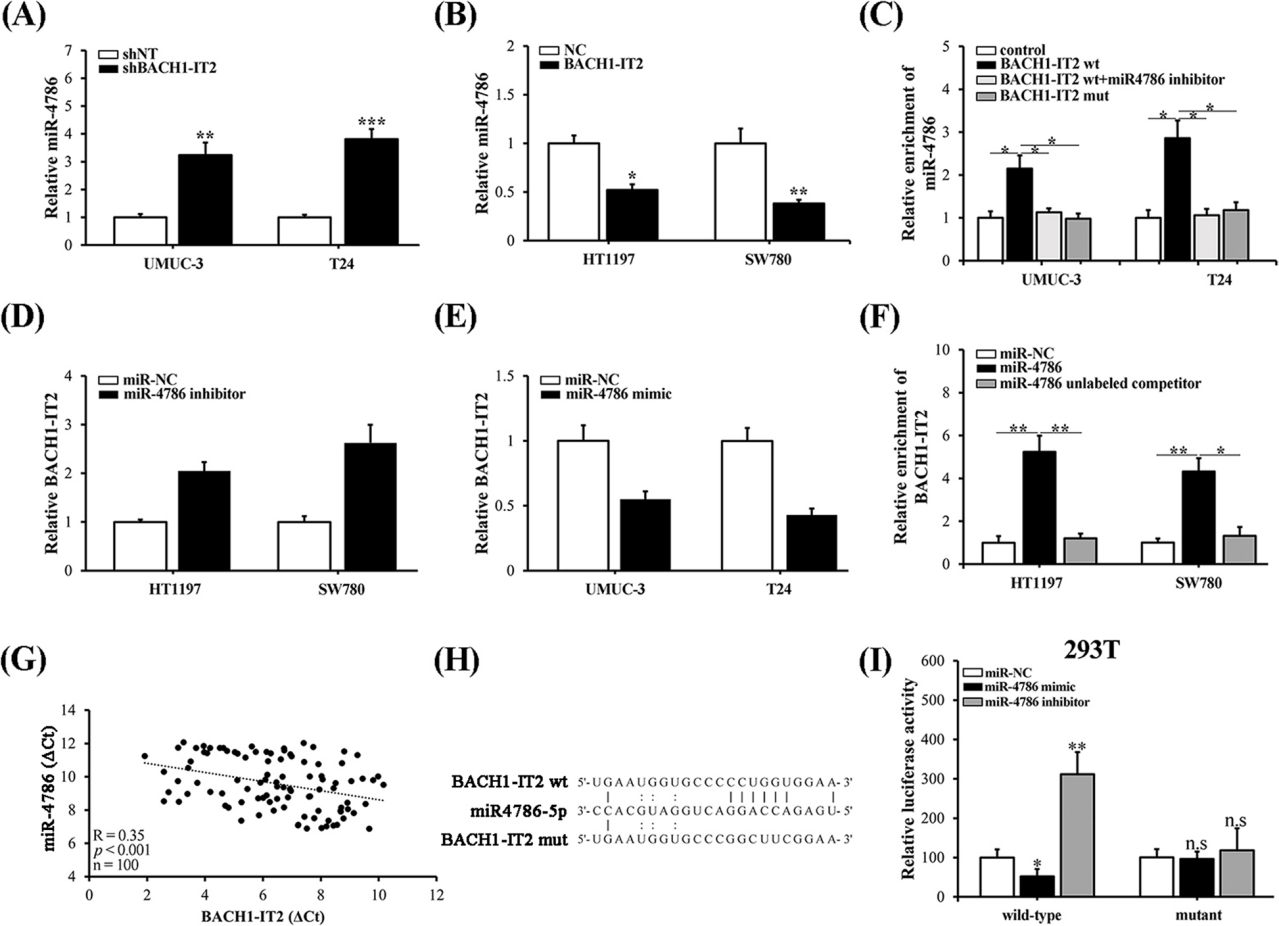
BACH1-IT2 functioned as ceRNA against miR-4786 in bladder cancer. **A** Relative expression of miR-4786 in both UMUC-3 and T24 cells transfected with either negative control or BACH1-IT2-specific shRNA. **B** Relative expression of miR-4786 in both HT1197 and SW780 cells transfected with either control or BACH1-IT2-expressing plasmids. **C** RNA-pulldown results in both UMUC-3 and T24 cells with control, wild-type LINC0973 fragment, or corresponding mutant, respectively. **D** Relative expression of BACH1-IT2 in both HT1197 and SW780 cells transfected with either negative control or miR-4786 inhibitors. **E** Relative expression of BACH1-IT2 in both UMUC-3 and T24 cells transfected with either control or miR-4786 mimics. **F** RNA-pulldown results in both HT1197 and SW780 cells with either control or miR-4786. **G** Reverse correlation analysis between endogenous miR-4786 and BACH1-IT2 in bladder tumor samples (*n* = 100). **H** Illustration of aligned miR-4786 with both wild-type and mutated BACH1-IT2. **I** Luciferase reporter results in 293 T cells co–transfected with BACH1-IT2 (wt and mut) and miR-NC, miR-4786 mimic, and miR-4786 inhibitor, respectively

To coordinate the divergent roles of BACH1-IT2 and miR-4786 in regulation of Siglec-15 in bladder cancer, we therefore analyzed the potentially regulatory effects between these two molecules. As presented in Fig. 5A and B, miR-4786 was remarkedly increased in BACH1-IT2-deficient UMUC-3 and T24 cells, and decreased in BACH1-IT2-proficient HT1197 and SW780 cells. Notably, miR-4786 was significantly enriched by BACH1-IT2 transcript in both UMUC-3 and T24 cell, which could be abolished by either co-administration of miR-4786 inhibitor or mutations destroying interaction between miR-4786 and BACH1-IT2 (Fig. 5C). Similarly, BACH1-IT2 transcripts were remarkedly up-regulated by miR-4786 inhibitor in both HT1197 and SW780 cells and down-regulated by miR-4786 mimic (Fig. 5D, E). RNA pulldown assay performed with miR-4786 tremendously enriched BACH1-IT2 transcripts in HT1197 and SW780 cells as well (Fig. 5F), which was completely inhibited by introduction of competitor miRs. The negative correlation between miR-4786 and BACH1-IT2 in bladder tumor panel was presented in Fig. 5G. Furthermore, direct interaction and regulation between BACH1-IT2 and miR-4786 was interrogated by luciferase reporter assay. Alignment between primary sequence of miR-4786 and putative recognizing region of BACH1-IT2 was illustrated in Fig. 5H. Co-transfection of 293 T cells with miR-4786 mimic significantly inhibited BACH1-IT2-fused luciferase activities, while miR-4786 inhibitor oppositely intensified luciferase signal (Fig. 5I). These regulatory effects were completely abolished by mutation introduced to impair interaction between miR-4786 and BACH1-IT2. Taken together, our data suggested the ceRNA role of BACH1-IT2 against miR-4786, which consequently contributed to Siglec-15 regulation in bladder cancer.

### BACH1-IT2-miR-4786-Siglec-15 axis involved in immune modulation in bladder cancer

**Fig. 6:**
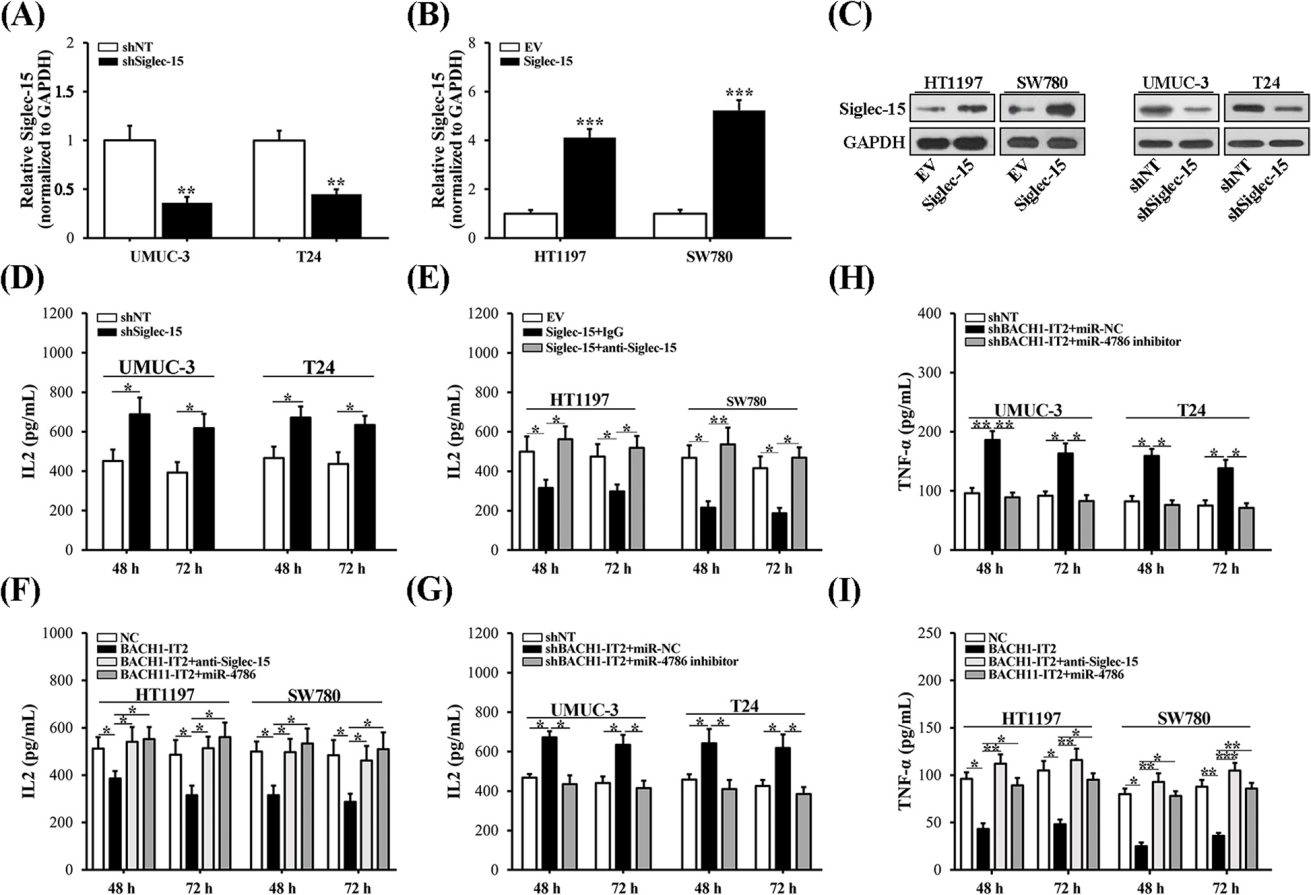
BACH1-IT2-miR-4786-Siglec-15 axis involved in immune activations in bladder cancer cells. **A** Establishment of Siglec-15 knockdown cells in UMUC-3 and T24 was confirmed by real-time PCR. **B** Establishment of Siglec-15 overexpression cells in HT1197 and SW780 cells was confirmed by real-time PCR. **C** Western blot results of relative expression of Siglec-15 protein in cells described in A and B, and the croppted blots were presented correspondingly. **D** Interleukin-2 (IL-2) production analysis in Jurkat: UMUC-3/T24 (shNT or shSiglec-15, 2:1) co–culture system. IL-2 was quantified with an ELISA kit at 48 and 72 h, respectively. **E** IL-2 production analysis in Jurkat: HT1197/SW780 (EV or Siglec-15, 2:1) co–culture system. **F** IL-2 production analysis of Jurkat co–culture system with both HT1197 and SW780 cells transfected with control or BACH1-IT2 in combination with either miR-NC or miR4786 mimics. **G** IL-2 production analysis of Jurkat co–culture system with both UMUC-3 and T24 cells transfected with negative control or shBACH1-IT2 in combination with either miR-NC or miR-4786 inhibitor. **H** Tumor necrosis factor-α (TNF-α) secretion analysis of Jurkat co–culture system with both UMUC-3 and T24 cells transfected with negative control or shBACH1-IT2 in combination with either miR-NC or miR-4786 inhibitors. **I** TNF-α secretion analysis of Jurkat co–culture system with both HT1197 and SW780 cells transfected with either control or BACH1-IT2 in combination with either miR-NC or miR-4786 mimics

In view of the critical roles of Siglec-15 in tumor immune modulation and our previous identified LINC00973-miR-7109-Siglec-15 regulatory axis in ccRCC, here we sought to clarify whether the similar scenario predominated in bladder cancer. To this end, we established both Siglec-15-deficient UMUC-3 and T24 cells and -proficient HT1197 and SW780 cells. The stable cell lines were validated by both quantitative PCR (Fig. 6A, B) and western blotting analysis (Fig. 6C). To evaluate the potential immune suppressive effects of Siglec-15 in bladder cancer, here we employed MART-I-specific 1D3 TCR-positive Jurkat cell to co-culture with MART-I peptide pre-loaded cancer cells. IL-2 secretion was stimulated by Siglec-15 deficiency (Fig. 6D) but suppressed by ectopic Siglec-15 overexpression, while the latter was alleviated by supplement with Siglec-15-specific antibodies (Fig. 6E). This observation was in agreement with our previous study in ccRCC. Next, we introduced BACH1-IT2 instead of Siglec-15 into both HT1197 and SW780, and significant immune inhibitory effects was noticed as well (Fig. 6F). However, co-administration with either Siglec-15 antibody or miR-4786 mimic notably alleviated this effect. In contrast, BACH1-IT2 silencing in UMUC-3 and T24 cells stimulated IL-2 secretion, which was abolished by simultaneous introduction of miR-4786 inhibitor (Fig. 6G). We further evaluated the immune activation in our system with secretory TNF-α. In line with BACH1-IT2-miR-4786-Siglec-15 regulatory axis mentioned before, combination of BACH1-IT2-deficiency with miR-4786 inhibitor reversed the stimulatory effect of BACH1-IT2-deficiency alone in TNF-α production (Fig. 6H). Similarly, either Siglec-15-specific antibody or miR-4786 were capable of restored TNF-α secretion, which was compromised by ectopic expression of BACH1-IT2 (Fig. 6I). The consistent while less significant results were noticed in regard to secretion of IL-4 and IL-10 as well (Fig. S3). Therefore, our data confirmed the important contribution of BACH1-IT2-miR-478 in immune suppressive function in bladder cancer.

## Discussion

The pioneering study by Wang et al. identified Siglec-15 as an important immune suppressor in multiple types of human cancer, which was exploited as potential target for normalizing cancer immunotherapy afterwards. The following study characterized the positivity of Siglec-15 in gastric cancer, which tightly associated with tumor advance [14]. Zheng et al. reported the activation of epithelial-mesenchymal transition and Beclin-1/ATG14 signaling by Siglec-15-induced autophagy in human osteosarcoma [15]. In glioma, dynamic expression of Siglec-15 in peritumoral macrophages was acknowledged to confer immunosuppressive microenvironment [16]. Our previous data reported LINC00973 as positive regulator of cell surface Siglec-15 in ccRCC, which exerted its pro-tumoral role via sponging miR-7109. Here we presented similar scenario in human bladder cancer, wherein BACH1-IT2 and miR-4786 were competitively involved in Siglec-15 regulation. The aberrant high-level of BACH1-IT2 in bladder cancer significantly inhibited immune activation in our in vitro co-culture system as indicated by secretion of both IL-2 and TNF-α. The immune suppressive effects imposed by BACH1-IT2 were readily antagonized by miR-4786, which notably highlighted the therapeutic potential of this small molecular RNA. Summarily, we elucidated the molecular mechanism underlying Siglec-15 regulation in human bladder cancer by BACH1-IT2-miR-4786, which consequently involved in immune modulation in this disease.

Despite the increasing archival of miRs in number of human cancers [17], the pathophysiological characteristics of miR-4786 was little addressed so far. Zhu et al. identified a 3-miRs signature in both pediatric and adolescent cytogenetically normal acute myeloid leukemia, wherein miR-4786 was shown associating with favorable prognosis [18]. Here we bioinformatically predicted and experimentally validated that Siglec-15 as direct target of miR-4786, which was also subjected to competition with BACH1-IT2. Our data highlighted the anti-tumoral properties of miR-4786 in human bladder cancer, which efficiently stimulated immune activation in tumor-immune cell co-culture system. In this regard, our observation implicated a great potential of miR-4786 for either therapeutic exploitation or prognostic application.

The lncRNA BACH1-IT2 located in the intronic region of BACH1-IT2 gene which encoded a transcription factor belonging to the cap ‘n’collar type of basic region leucine zipper factor family (CNC-bZip). Whether BACH1-IT2 affected its host gene transcription or splicing through complementary duplex was still to be addressed. The physiologic role of BACH1-IT2 was less recognized and risky prognostic link to soft-tissue sarcoma was reported along with other nine lncRNAs [19]. In line with this observation, here we demonstrated high abundance of BACH1-IT2 in bladder cancer, which was in concert with suppressed miR-4786, significantly contributed to high cell surface Siglec-15. The regulatory axis along BACH1-IT2-miR-4786-Siglec-15 consequently established immune suppression and evasion, and clinically linked to unfavorable outcomes. In addition to therapeutic Siglec-15 antibody, our data suggested the great promise of any combinational targeting BACH1-IT2-miR-4786-Siglec-15 signaling for clinical interventions. The intrinsic issues of combinational therapy such as off-target might pose an impediment to clinical management, which made the optimal dosage and timing of combination critically important during in vitro and animal trials.

In summary, here we characterized high level of Siglec15 in human bladder cancer, which was relatively predictive of both tumor stages and overall prognosis. We further identified BACH1-IT2, which competitively sponging miR-4786, therefore positively involved in Siglec-15. The uncovered BACH1-IT2-miR-4786-Siglec-15 axis in bladder cancer phenotypically forged an immune suppressive microenvironment, which consequently favored immune evasion of tumor cell. Our results not only offered insightful understanding the importance of BACH1-IT2-miR-4786-Siglec-15 in bladder cancer, but also proposed the promising opportunity for therapeutic exploitations. In this regard, our study potentially benefited both bladder cancer research and clinical treatments.

### Supplementary Information

**Supplementary Material 1. **

**Supplementary Material 2. **

## Data Availability

Data is provided within the manuscript or supplementary information files.
