# Supplementary material for: Identification of BACH1-IT2-miR-4786-Siglec-15 immune suppressive axis in bladder cancer
Source: BMC Cancer. 2024 Mar 11;24:328. doi: 10.1186/s12885-024-12061-8 (PMC10926634; doi:10.1186/s12885-024-12061-8)

**Fig. S1. Representative IHC images of immunosuppressive markers in bladder tumor.** CD4 (rabbit antibody, 1:100, #25229, Cell Signaling Technology), CD33 (rabbit antibody, 1:200, MA5-44315, ThermoFisher) and CD204 (mouse antibody, 1:500, 14-9054-82, ThermoFisher) were stained in patients with high- and low- Siglec-15 abundance.


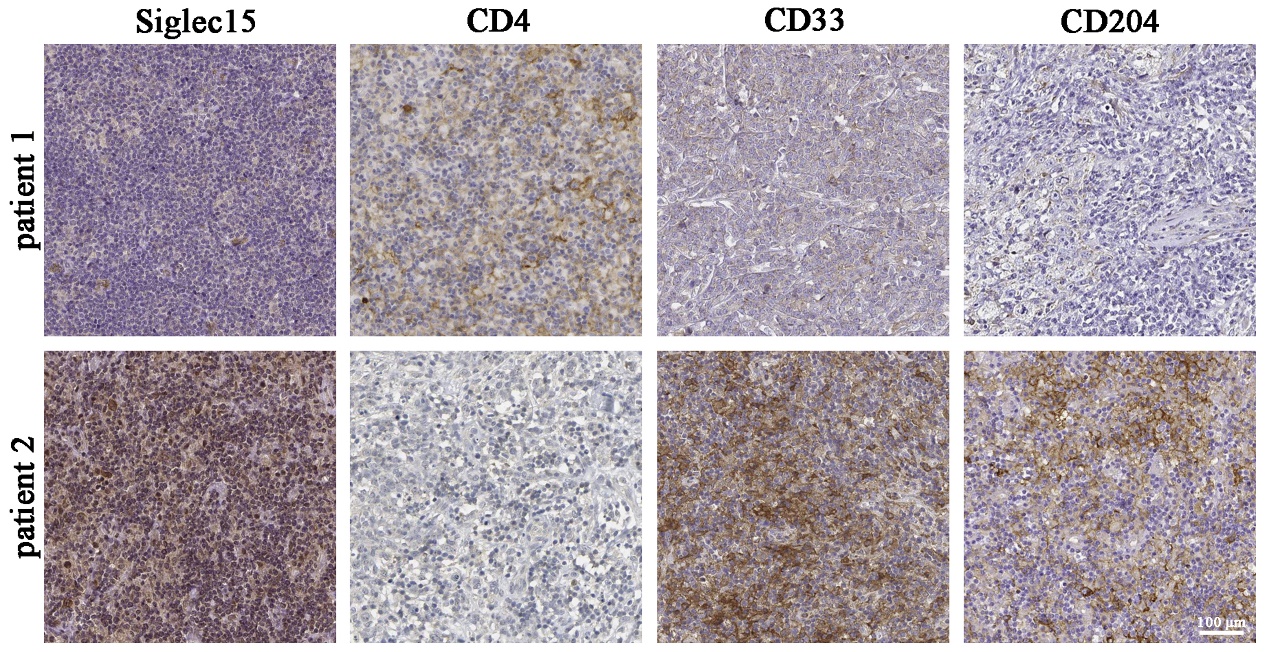


**Fig. S2. Quantitative analysis of western blotting results**. The intensity of the individual bands was quantified by densitometry and normalized to the corresponding input control (GAPDH). The compared samples were derived from the same experiment and loading controls were run on the same blot as well.


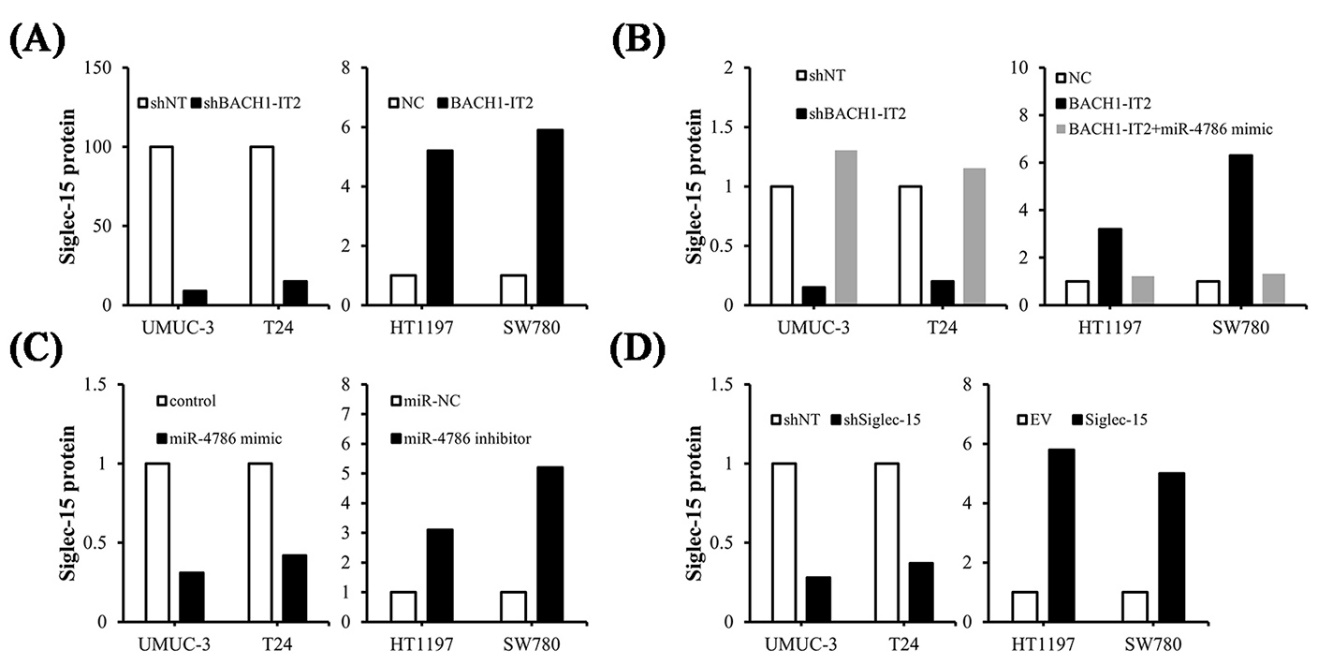


**Fig. S3. ELISA determination of Interleukin-4 and -10 secretion in Jurkat: bladder cancer cells co-culture system.** (A) IL-4 production analysis in Jurkat: UMUC-3 (shNT, shSiglec-15, shBACH1-IT2+miR-NC, shBACH1-IT2+miR-4786 inhibitor, 2:1) co–culture system. (B) IL-10 secretion analysis of Jurkat co–culture system with UMUC-3 cells transfected with shNT, shSiglec-15, shBACH1-IT2+miR-NC or shBACH1-IT2+miR-4786 inhibitor at a ratio of 2:1. (C) IL-4 production analysis in Jurkat: HT1197 (EV, Siglec-15+IgG, Siglec-15+anti-Siglec-15, BACH1-IT2, BACH1-IT2+anti-Siglec-15, BACH1-IT2+miR-4786, 2:1) co–culture system. (D) IL-10 secretion analysis of Jurkat co–culture system with HT1197 cells transfected with EV, Siglec-15+IgG, Siglec-15+anti-Siglec-15, BACH1-IT2, BACH1-IT2+anti-Siglec-15 or BACH1-IT2+miR-4786 at a ratio of 2:1. IL-4 and IL-10 was quantified with an ELISA kit at 48 and 72 h, respectively.


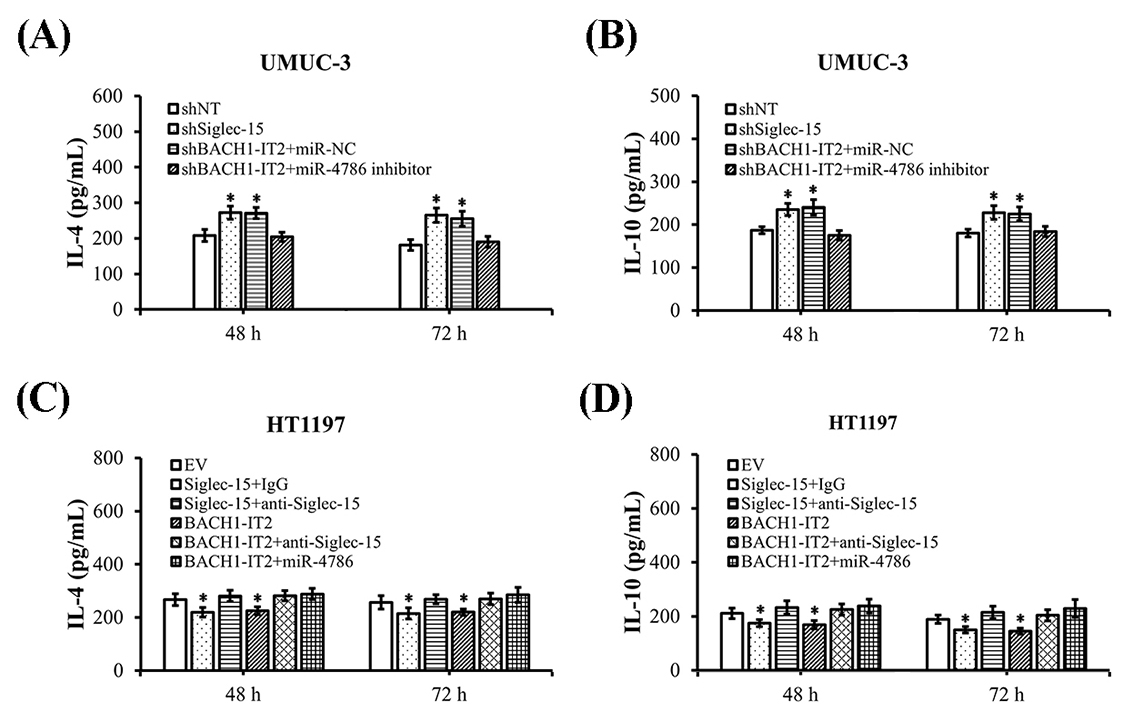

Supplement: Supplementary file 1 — Supplementary Material 1. [file 12885_2024_12061_MOESM1_ESM.docx]
