# Supplementary material for: Identification of BACH1-IT2-miR-4786-Siglec-15 immune suppressive axis in bladder cancer
Source: BMC Cancer. 2024 Mar 11;24:328. doi: 10.1186/s12885-024-12061-8 (PMC10926634; doi:10.1186/s12885-024-12061-8)

Figure 2F

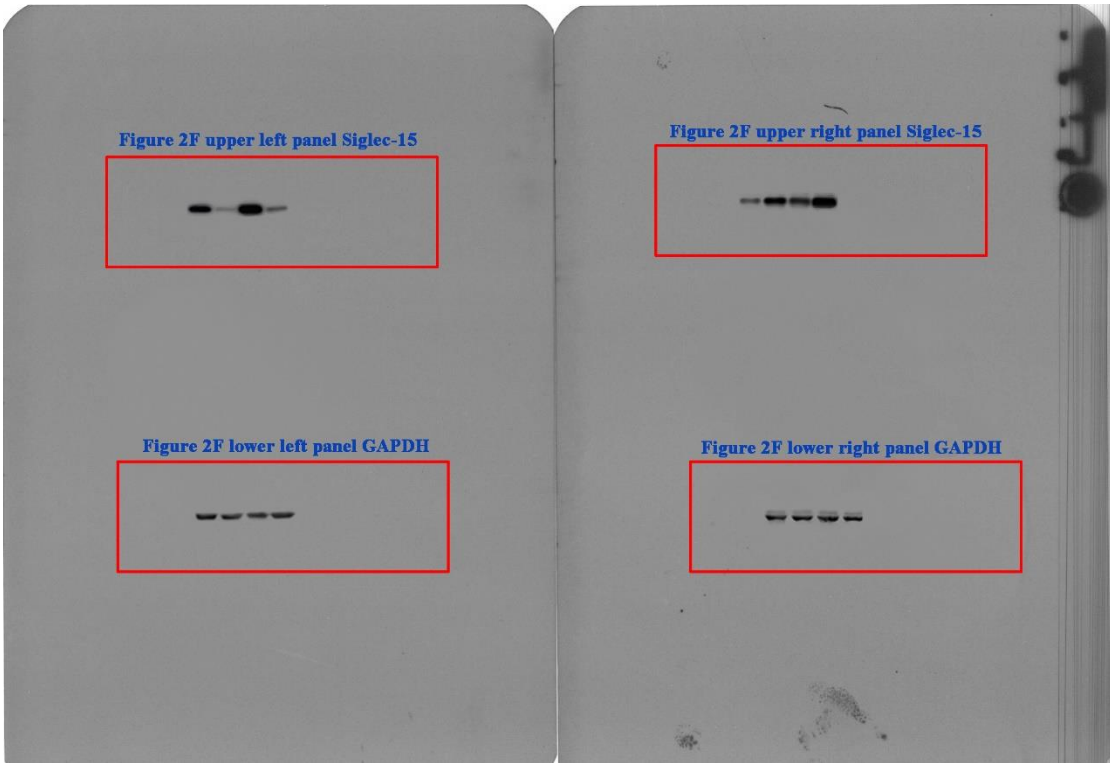
Figure 2F upper left panel Siglec-15
Figure 2F upper right panel Siglec-15
Figure 2F lower left panel GAPDH
Figure 2F lower right panel GAPDH

Figure 3C left panel

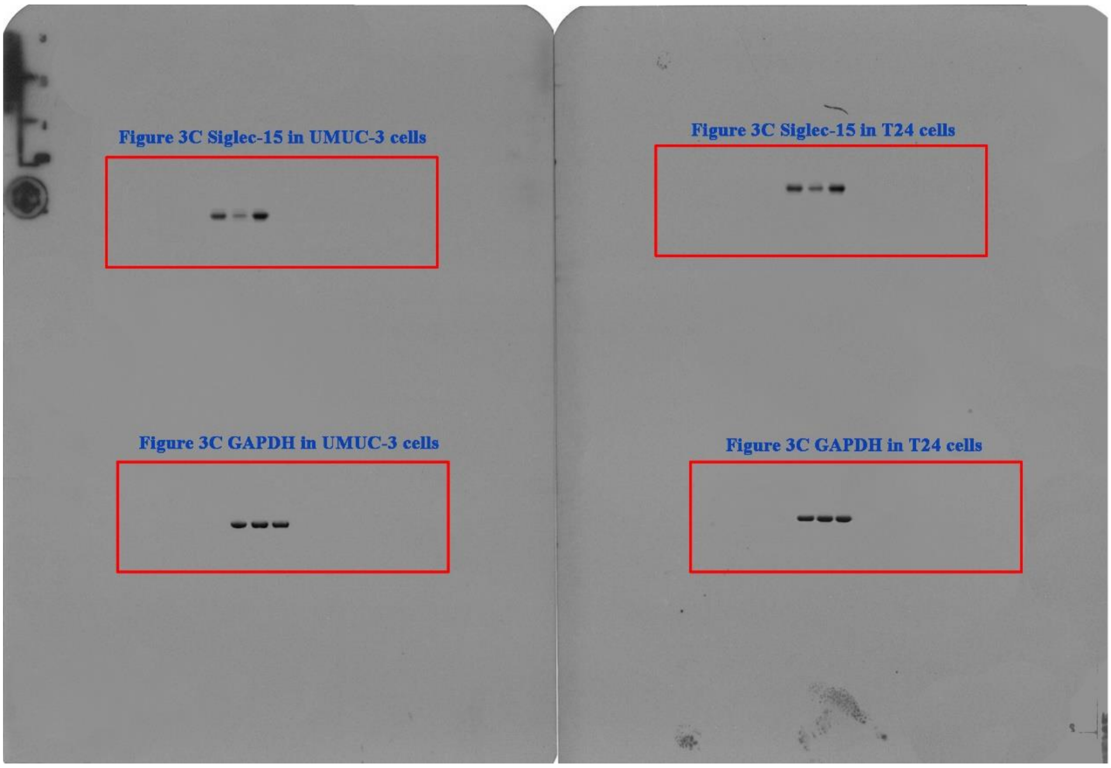

Figure 3C right panel

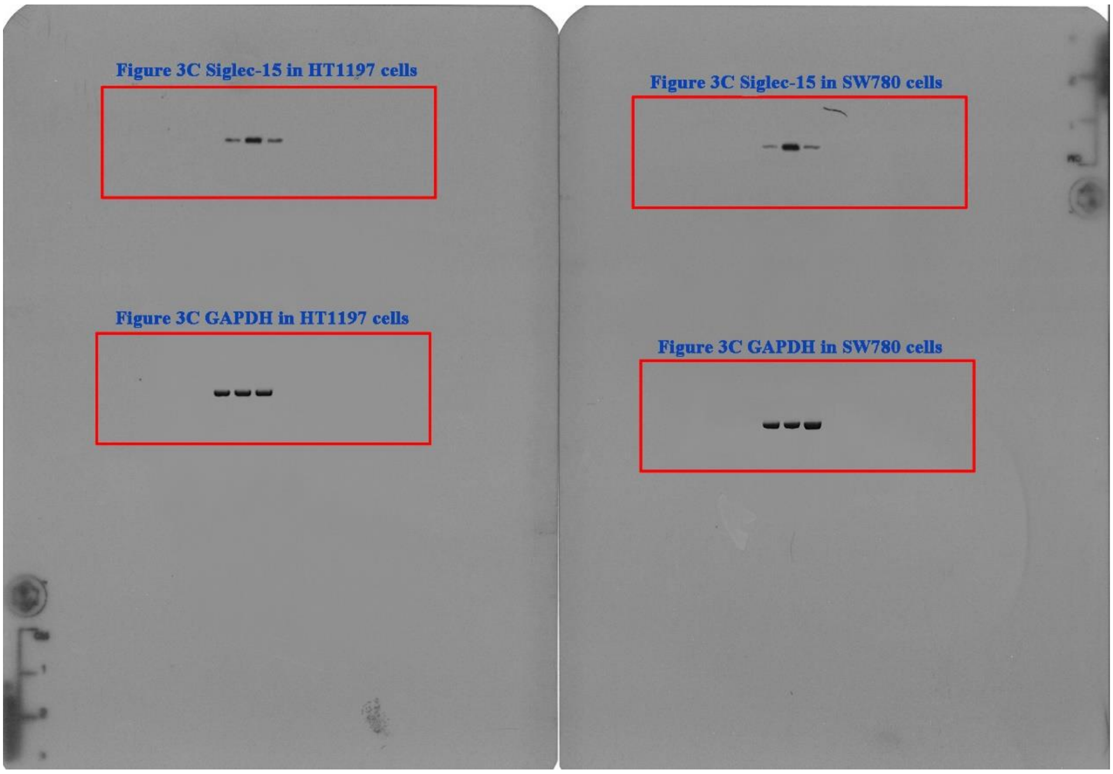

Figure 4E left panel

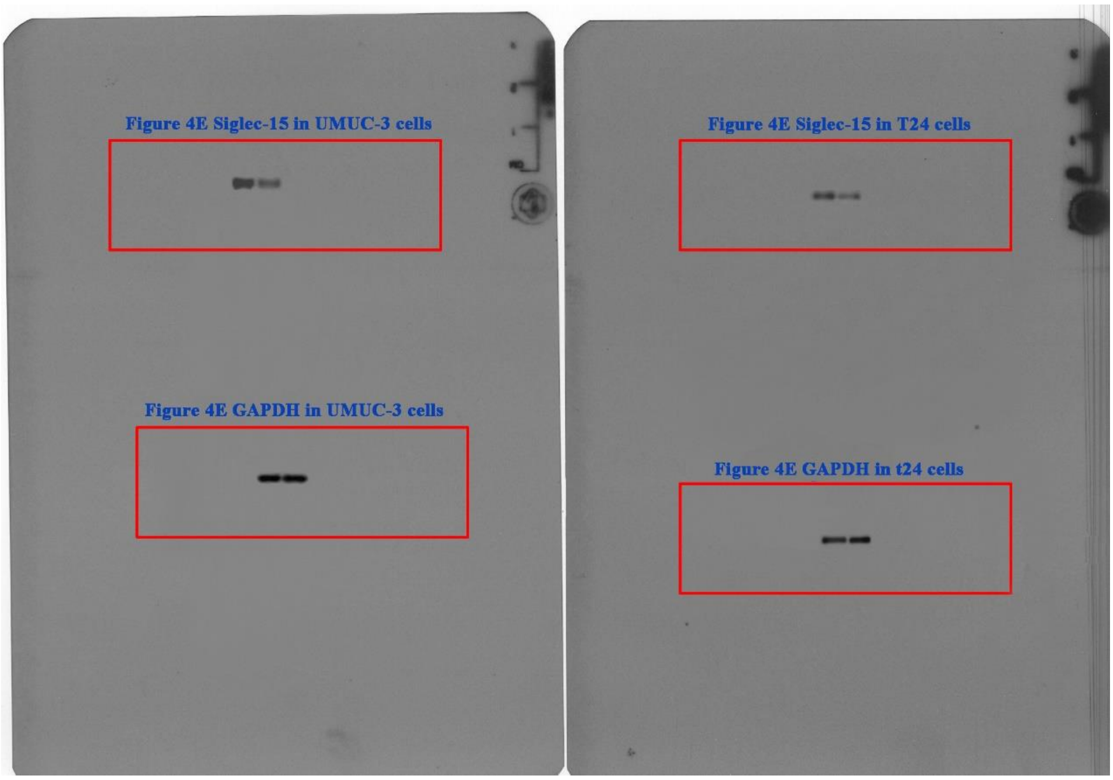

Figure 4E right panel

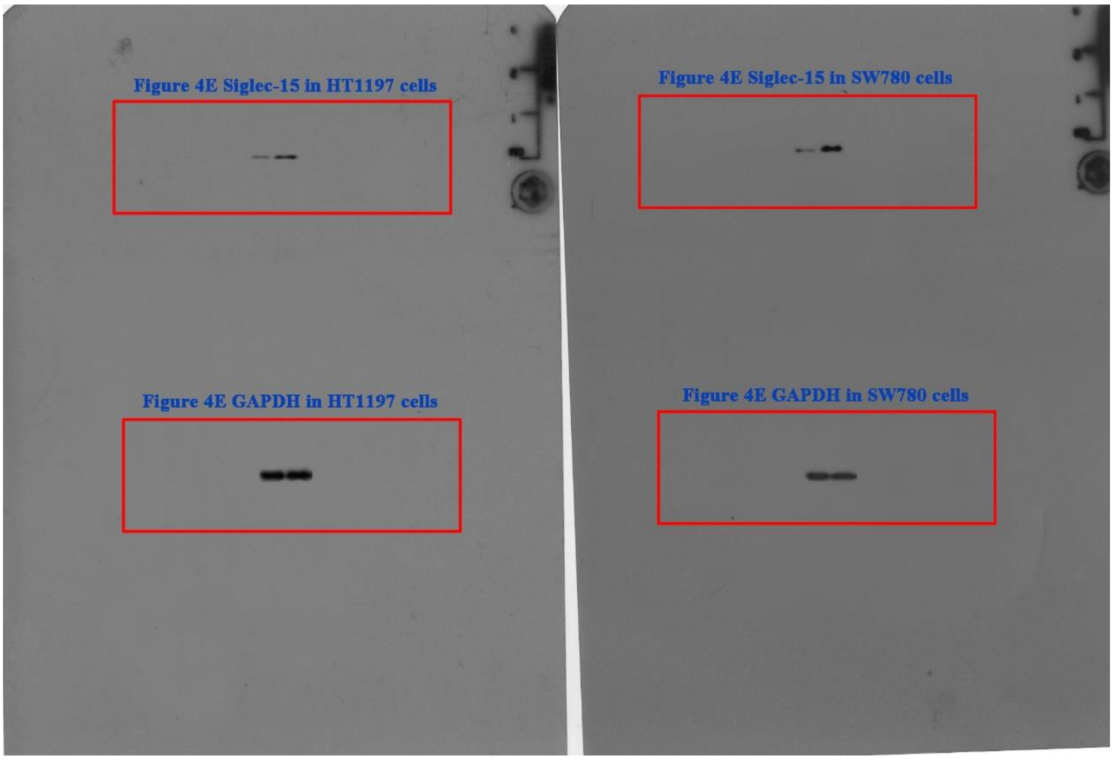

Figure 6C left panel

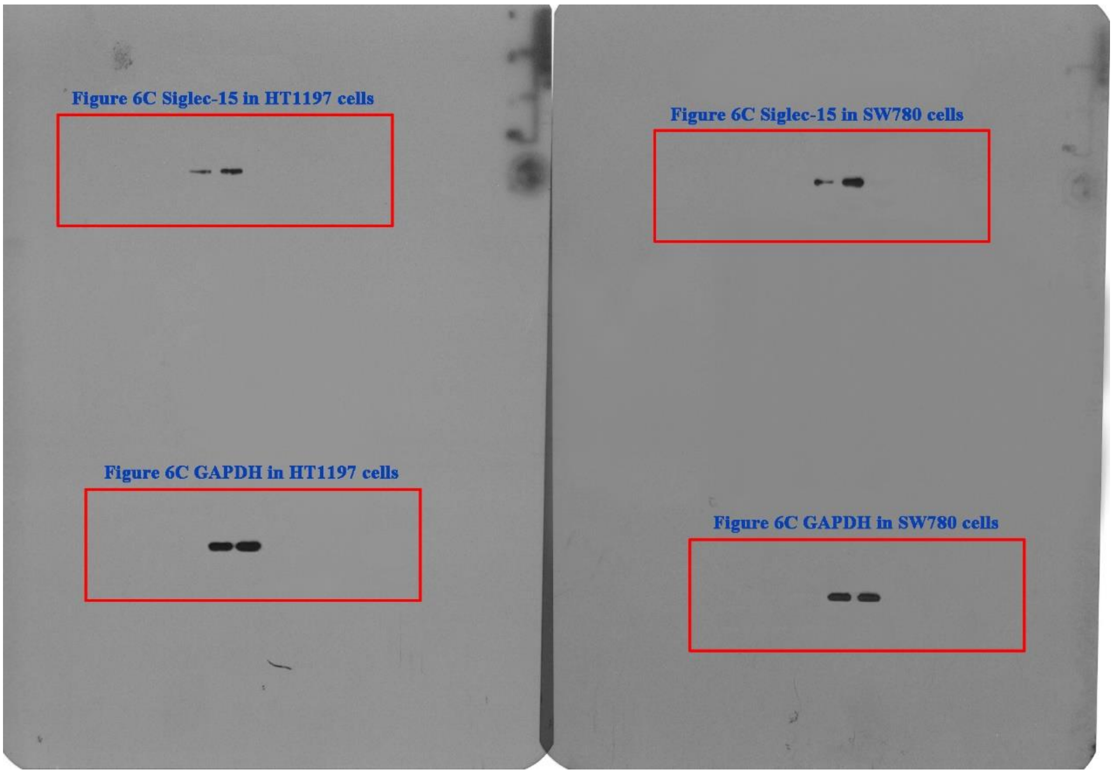

Figure 6C right panel

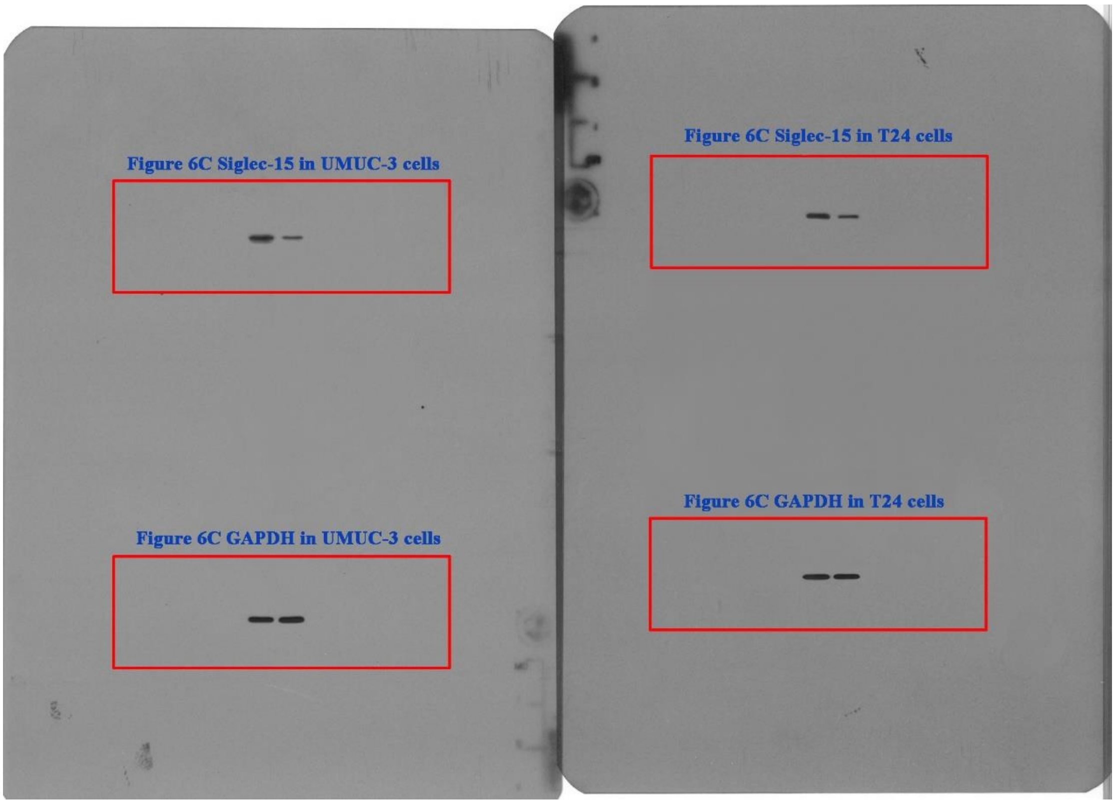

Supplement: Supplementary file 2 — Supplementary Material 2. [file 12885_2024_12061_MOESM2_ESM.pdf]
